# Supplementary material for: Heterogeneity in the gene regulatory landscape of leiomyosarcoma
Source: NAR Cancer. 2023 Jul 24;5(3):zcad037. doi: 10.1093/narcan/zcad037 (PMC10365024; doi:10.1093/narcan/zcad037)
Supplement: zcad037_Supplemental_Files [file zcad037_supplemental_files.zip › Supplementary_Figures_and_Tables_revised_15_05.pdf]

Supplemental Table S1. Proportions of pathways of different sizes among Reactome pathways and pathways identified with PORCUPINE in the TCGA-LMS and DKFZ-LMS datasets.

ptw genes	Reactome%	TCGA%	DKFZ%
<50	75.8	81.9	78
$\geqslant 50 \& \leqslant 100$	16	12.5	12
>100 & <150	5.9	4.2	5.8
$\geqslant 150$	2.3	1.4	4.4

Supplemental Table S2. Genes from the "E2F mediated regulation of DNA replication" pathway mapped to differential accessible regions (DARs) in each of the pairwise cell line comparisons.

Comparison	Among DARs	Not among DARs
SK-UT-1B vs SK-LMS-1	*PPP2R1B*, *PPP2R1A*, *E2F1*, *PRIM2*, *MCM8*, *TFDP2* *POLA2*	*PPP2CA*, *PRIM1*, *CCNB1*, *ORC4*, *POLA1*, *ORC1*, *PPP2CB*, *CDK1*, *ORC5*, *RB1*, *TFDP1*, *ORC2*
SK-UT-1B vs MES-SA	*PPP2R1A*, *ORC5*, *ORC1*, *CCNB1*, *ORC2*, *ORC4*, *RB1*, *PPP2R1B*	*POLA1*, *TFDP1*, *PRIM2*, *PPP2CA*, *PRIM1*, *TFDP2*, *POLA2*, *MCM8*, *PPP2CB*, *E2F1*, *CDK1*
SK-UT-1 vs SK-UT-1B	*CDK1*, *PRIM1*, *ORC4*	*CCNB1*, *PPP2CA*, *ORC2*, *TFDP2*, *PPP2R1A*, *ORC1* *TFDP1*, *PPP2R1B*, *POLA1*, *E2F1*, *RB1*, *ORC5*, *PPP2CB*, *PRIM2*, *POLA2*, *MCM8*
SK-UT-1 vs SK-LMS-1	*PPP2R1A*, *PPP2R1B*, *E2F1*, *PRIM2*, *MCM8*, *PRIM1* *PPP2CA*, *CCNB1*, *CDK1*, *POLA2*, *TFDP2*, *POLA1*	*TFDP1*, *ORC4*, *PPP2CB*, *ORC5*, *ORC2*, *RB1*, *ORC1*
SK-UT-1 vs MES-SA	*CCNB1*, *PPP2R1A*, *ORC4*, *ORC1*, *ORC2*, *ORC5*, *PRIM1*, *CDK1*, *PPP2R1B*, *TFDP1*, *POLA1*, *RB1*, *PRIM2*	*POLA2*, *PPP2CA*, *PPP2CB*, *MCM8*, *E2F1*, *TFDP2*
MES-SA vs SK-LMS-1	*PPP2R1B*, *PRIM2*, *E2F1*, *ORC5*, *MCM8*, *ORC1* *ORC4*, *ORC2*, *POLA2*, *PPP2CA*, *PPP2R1A*, *RB1*, *TFDP2*, *CCNB1*	*TFDP1*, *POLA1*, *CDK1*, *PRIM1*, *PPP2CB*

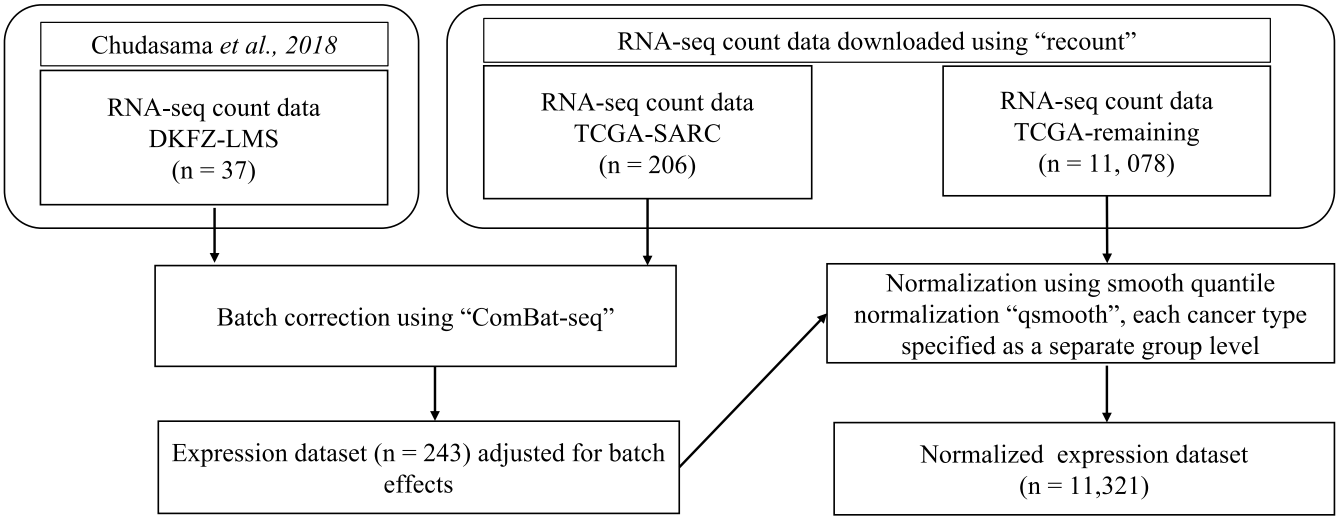

Supplementary Figure S1. Preprocessing and normalization workflow.

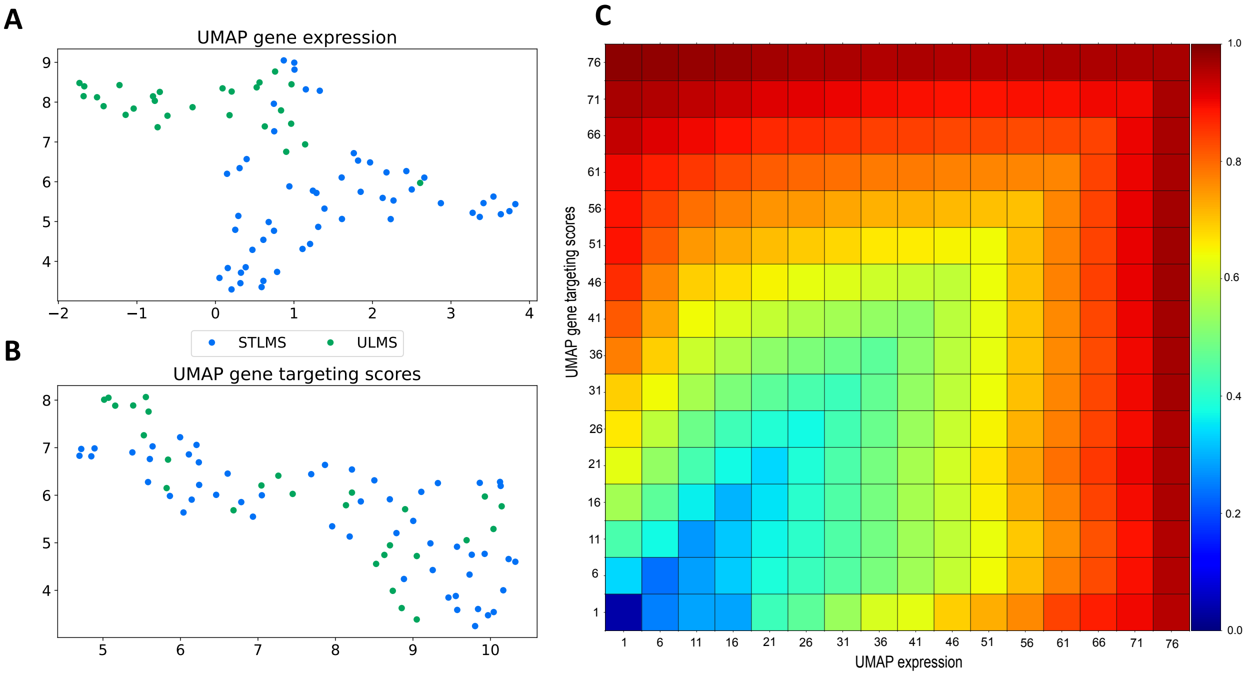

Supplementary Figure S2. UMAP visualization of the distribution of 80 TCGA leiomyosarcoma samples based on A. expression. B. gene targeting scores. C. Comparison between the two embeddings (UMAP on expression and UMAP on gene targeting scores). The axis depicts the number of nearest neighbours. The colour depicts the similarity scores, reflecting the proportion of overlapping neighbouring samples. STLMS: soft tissue leiomyosarcoma, ULMS: uterine leiomyosarcoma.

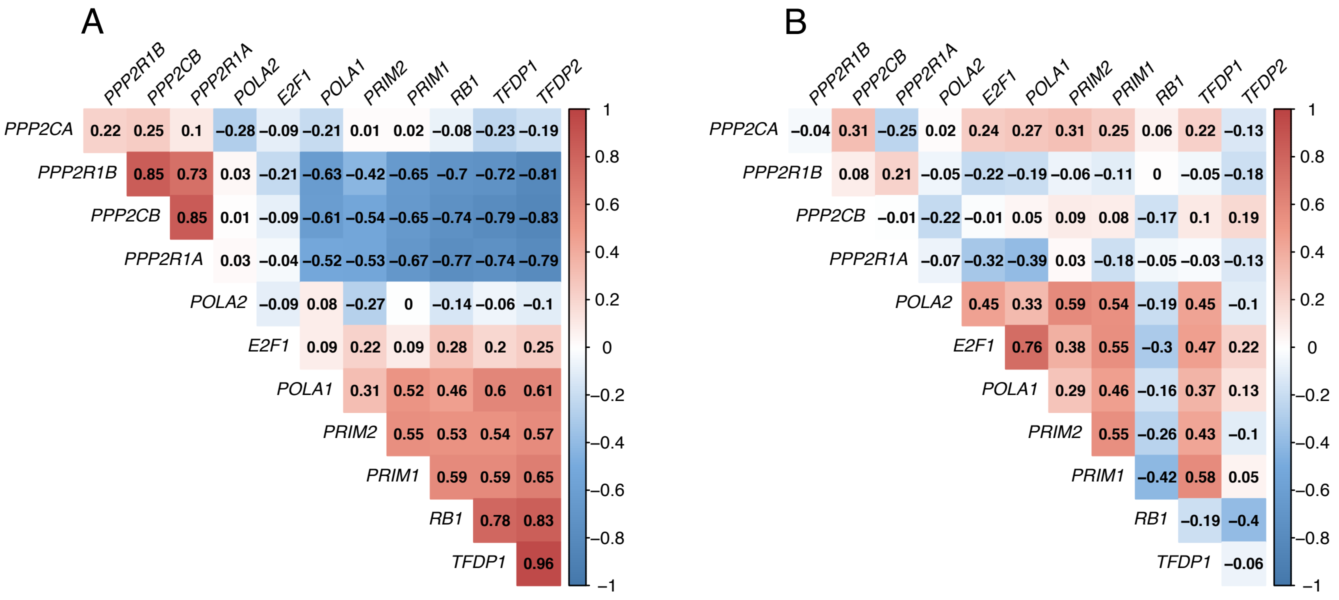

Supplementary Figure S3. Pearson correlations between A. targeting scores and B. expression levels of genes belonging to the pathway "Inhibition of replication initiation of damaged DNA by RB1/E2F1."

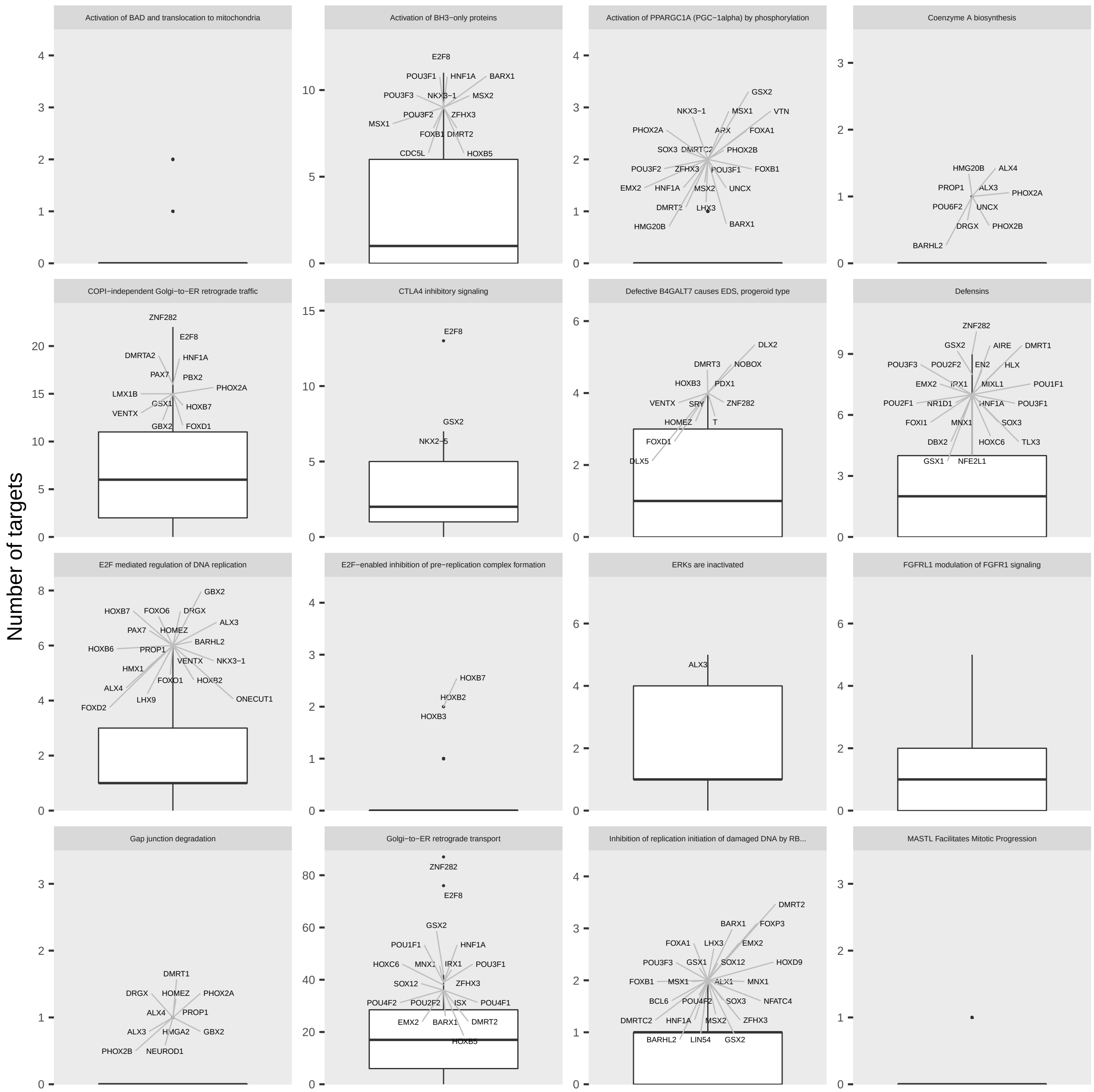

Number of targets
Activation of BAD and translocation to mitochondria
Activation of BH3−only proteins
E2F8
POU3F1
HNF1A
BARX1
POU3F3
NKX3−1
MSX2
POU3F2
ZFHX3
MSX1
FOXB1
DMRT2
CDC5L
HOXB5
Activation of PPARGC1A (PGC−1alpha) by phosphorylation
GSX2
NKX3−1
MSX1
VTN
PHOX2A
ARX
FOXA1
SOX3
DMRTC2
PHOX2B
POU3F2
ZFHX3
POU3F1
FOXB1
EMX2
HNF1A
MSX2
UNCX
DMRT3
LHX3
HMG20B
BARX1
Coenzyme A biosynthesis
HMG20B
ALX4
PROP1
ALX3
PHOX2A
POU6F2
UNCX
DRGX
PHOX2B
BARHL2
COPI−independent Golgi−to−ER retrograde traffic
ZNF282
E2F8
DMRTA2
HNF1A
PAX7
PBX2
PHOX2A
LMX1B
GSX1
HOXB7
VENTX
GBX2
FOXD1
CTLA4 inhibitory signaling
E2F8
GSX2
NKX2−5
Defective B4GALT7 causes EDS, progeroid type
DLX2
DMRT3
NOBOX
HOXB3
PDX1
VENTX
SRY
ZNF282
HOMEZ
T
FOXD1
DLX5
Defensins
ZNF282
GSX2
AIRE
DMRT1
POU3F3
POU2F2
EN2
HLX
EMX2
IPX1
MIXL1
POU1F1
POU2F1
NR1D1
HNF1A
POU3F1
FOXI1
MNX1
SOX3
DBX2
HOXC6
TLX3
GSX1
NFE2L1
E2F mediated regulation of DNA replication
GBX2
HOXB7
FOXO6
DRGX
ALX3
PAX7
HOMEZ
BARHL2
HOXB6
PROP1
VENTX
NKX3−1
HMX1
ALX4
FOXO1
HOXB2
ONECUT1
FOXD2
LHX9
E2F−enabled inhibition of pre−replication complex formation
HOXB7
HOXB2
HOXB3
ERKs are inactivated
ALX3
FGFRL1 modulation of FGFR1 signaling
Gap junction degradation
DMRT1
DRGX
HOMEZ
PHOX2A
ALX4
PROP1
ALX3
HMGA2
GBX2
PHOX2B
NEUROD1
Golgi−to−ER retrograde transport
ZNF282
E2F8
GSX2
POU1F1
HNF1A
HOXC6
MNX1
IRX1
POU3F1
SOX12
ZFHX3
POU4F2
POU2F2
ISX
POU4F1
EMX2
BARX1
DMRT2
HOXB5
Inhibition of replication initiation of damaged DNA by RB...
DMRT2
BARX1
FOXP3
FOXA1
LHX3
EMX2
POU3F3
GSX1
SOX12
HOXD9
FOXB1
MSX1
ALX1
MNX1
BCL6
POU4F2
SOX3
NFATC4
DMRTC2
HNF1A
MSX2
ZFHX3
BARHL2
LIN54
GSX2
MASTL Facilitates Mitotic Progression

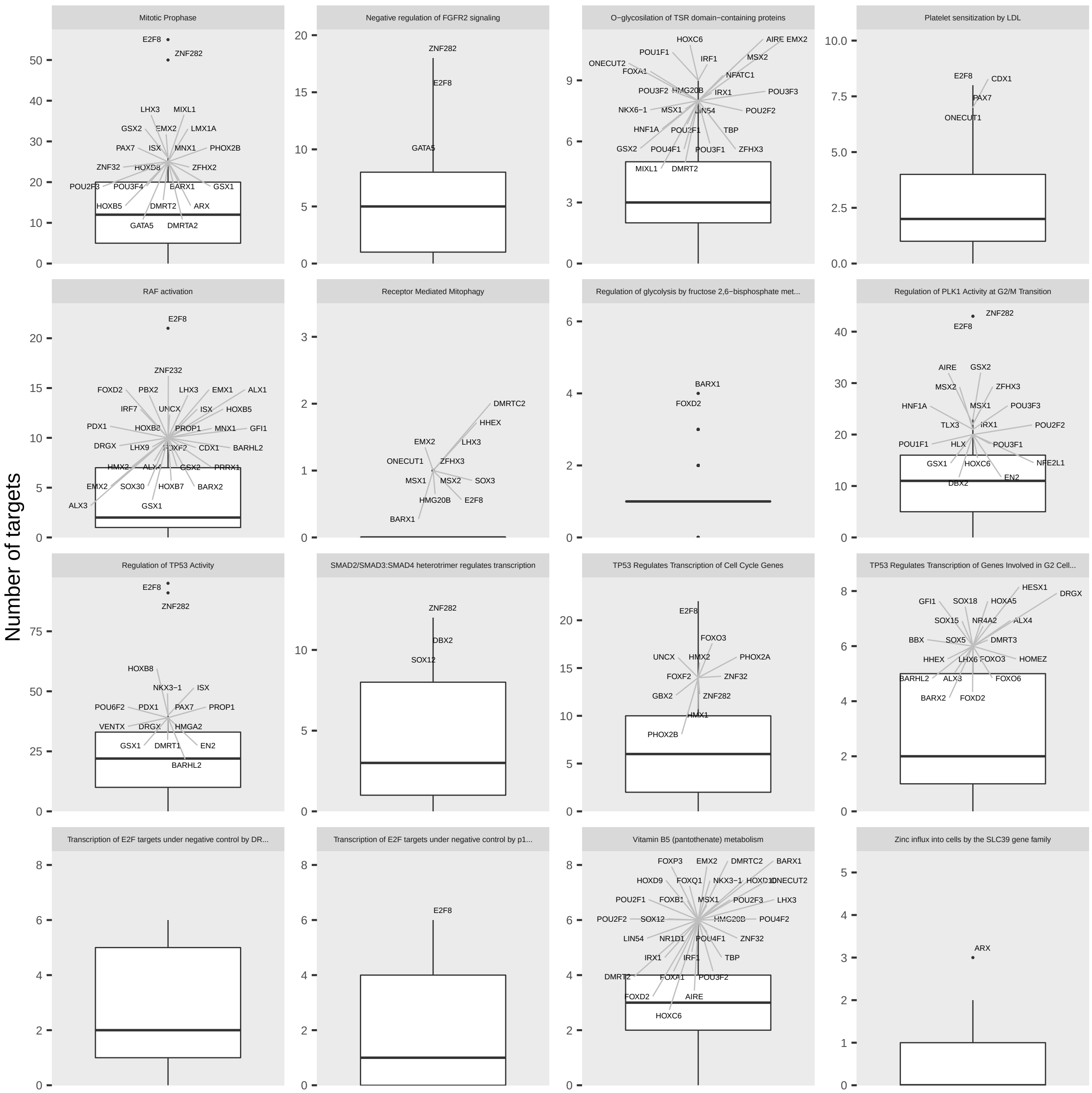

Supplementary Figure S4. Boxplots showing the number of targets for TFs with most highly weighted values to PC1 in each pathway. 32 out of 37 pathways had edge weights with contribution scores above the threshold. TFs with a number of targets greater than the 95th percentile are labelled.

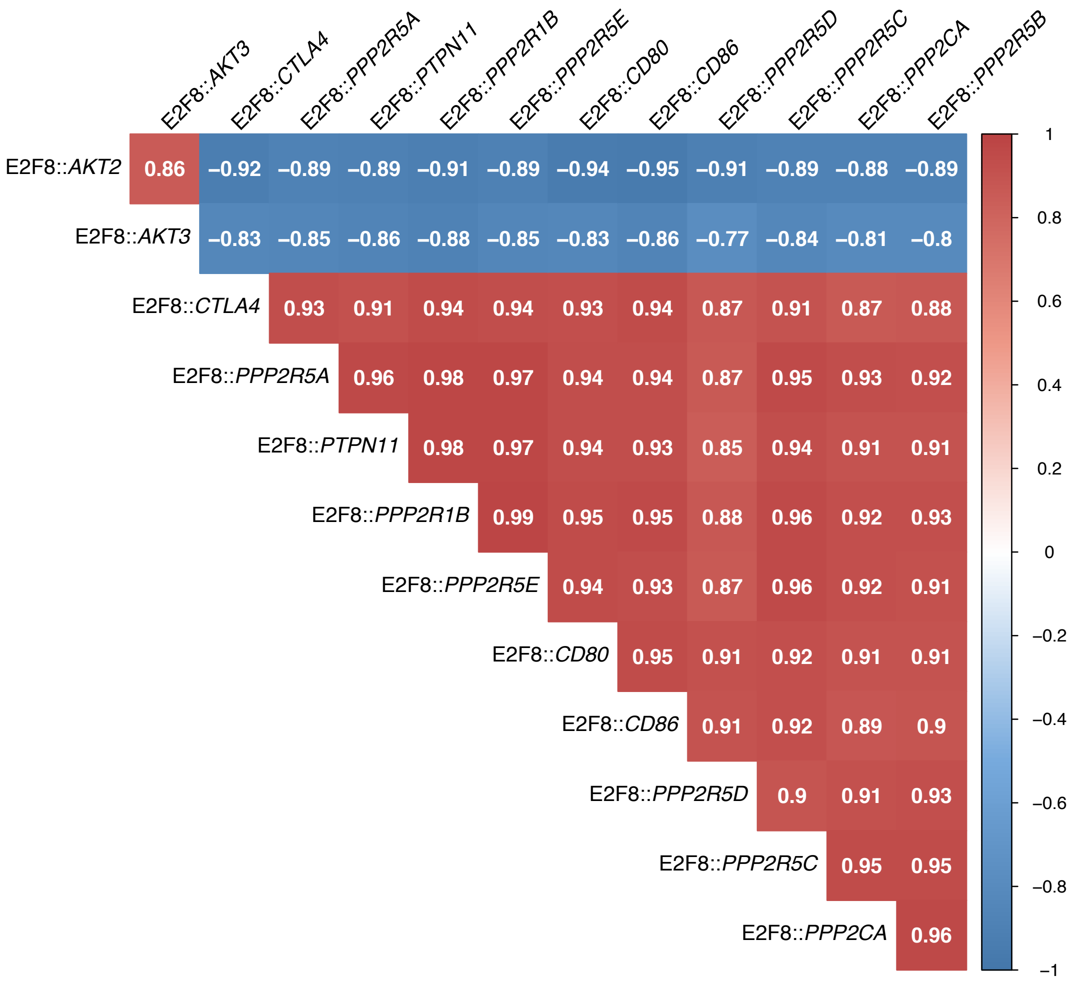

Supplementary Figure S5. Pearson correlations among edge weights of target genes of the transcription factor "E2F8" in the pathway "CTLA4 inhibitory signalling."

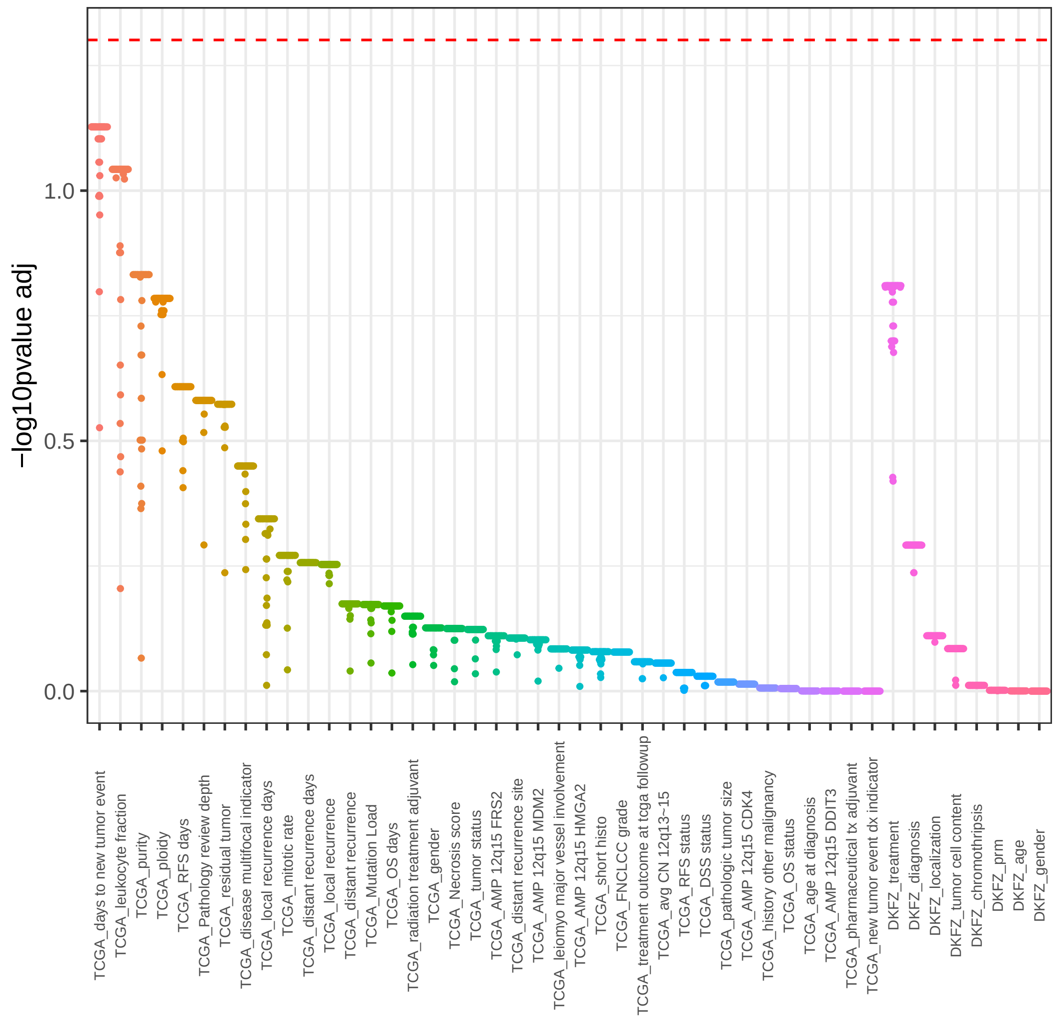

Supplementary Figure S6. Association of the clinical features of patients and pathway-based patient heterogeneity scores on PC1 in each of the 37 pathways. Associations are shown for both cohorts (TCGA and DKFZ). The y-axis indicated the negative log base 10 of the FDR-adjusted p-value. The dotted line indicates a p-value of 0.05.

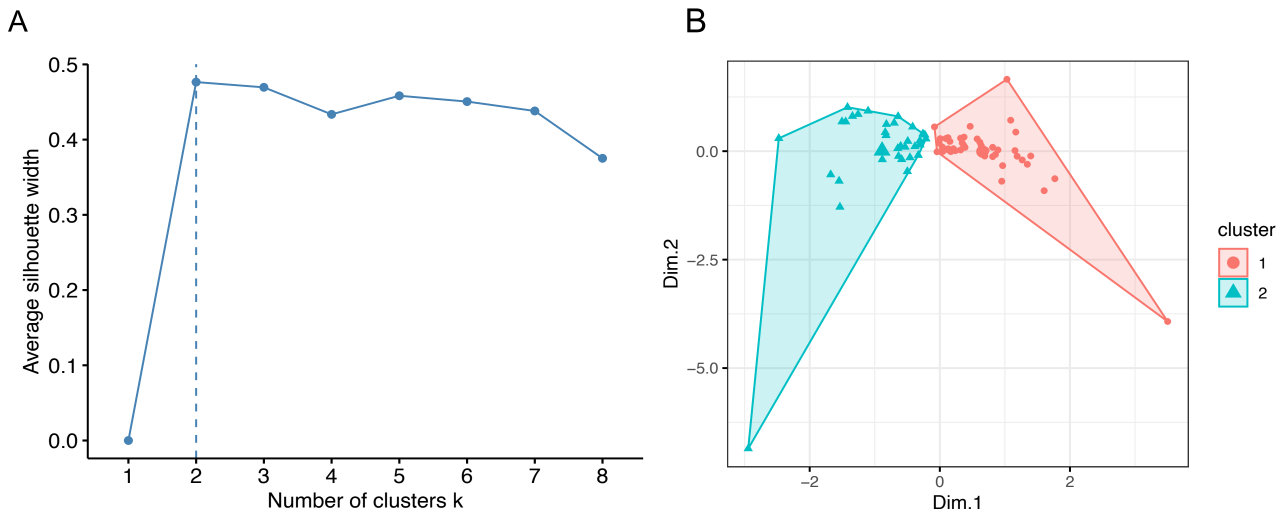

Supplementary Figure S7. K-means clustering of individual gene regulatory networks from the TCGA-LMS dataset based on the "E2F mediated regulation of DNA replication" pathway A. Identification of optimal number of clusters with the Average Silhouette Method. B. K-means cluster plot

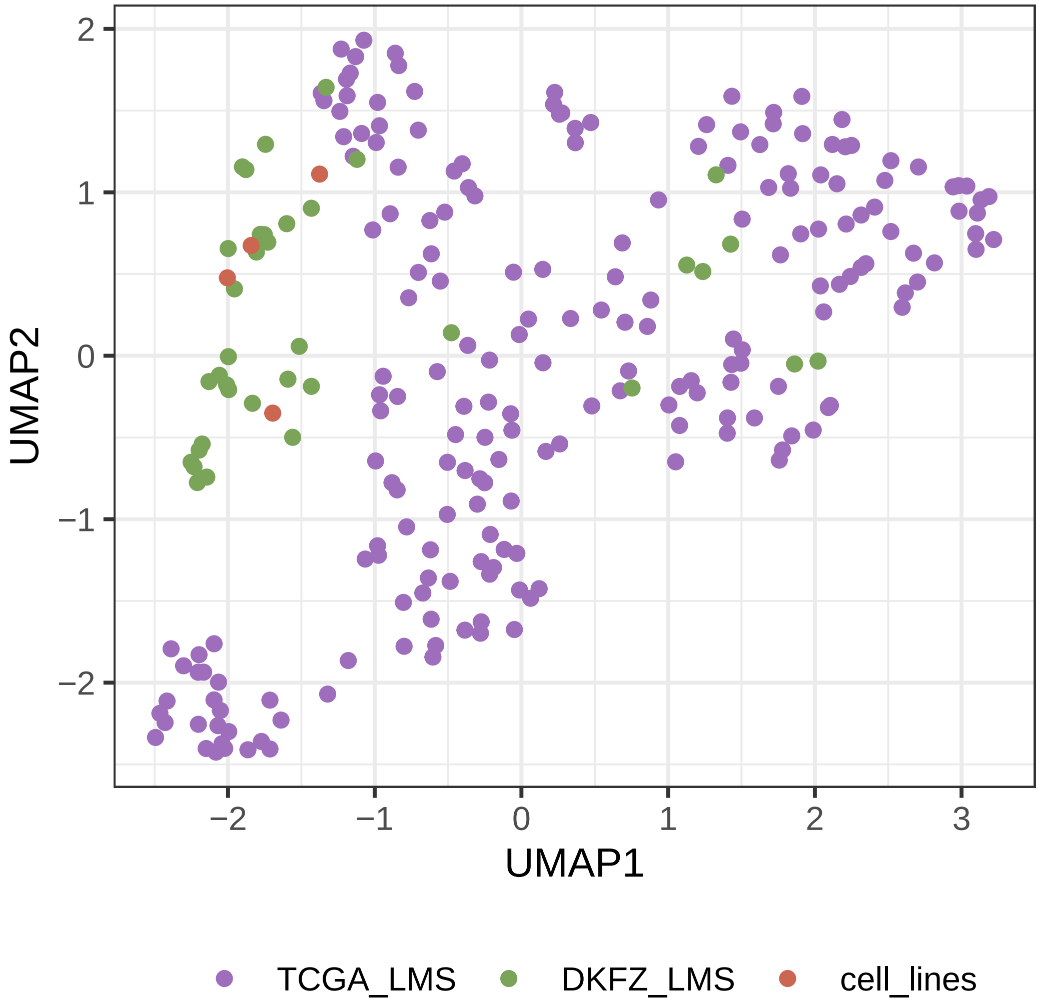

Supplementary Figure S8. UMAP visualization of the distribution of leiomyosarcomas from three different datasets (indicated with different colors).

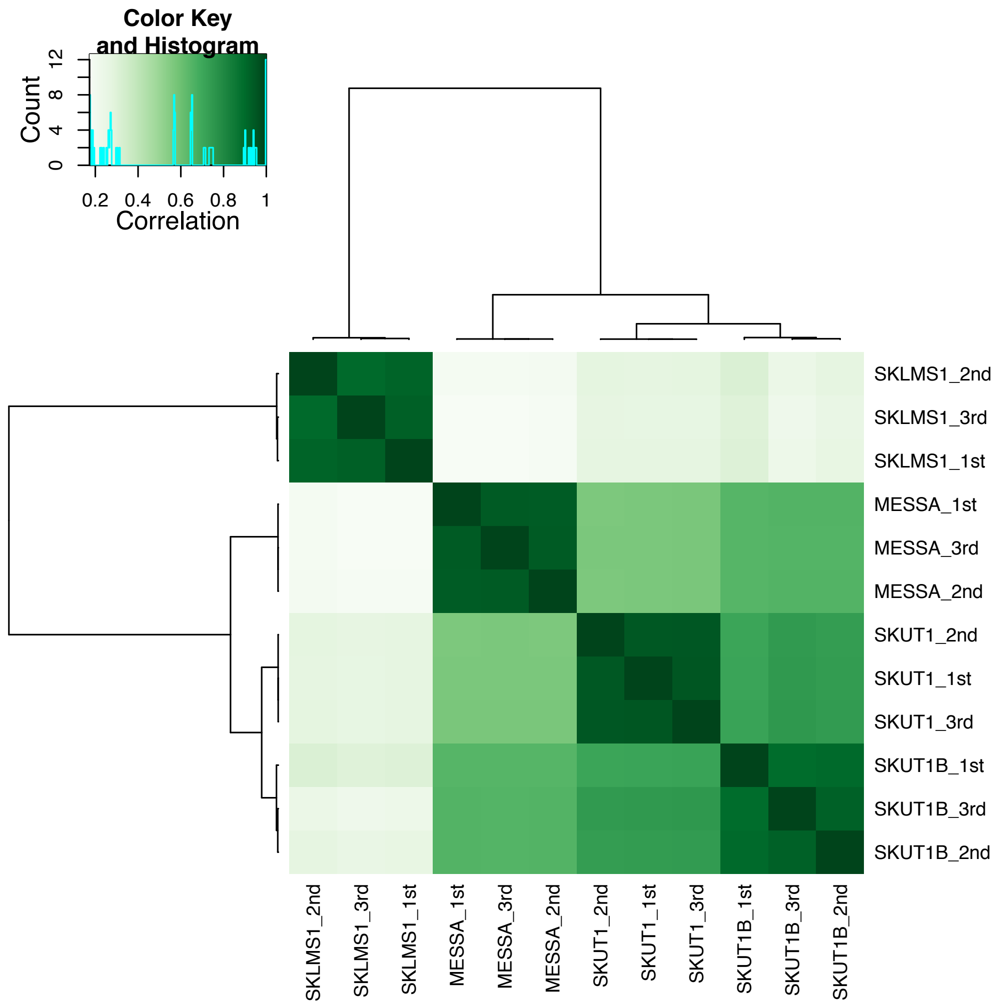

Supplementary Figure S9. Hierarchical clustering of cell lines based on their ATAC-seq profiles. Numbers indicate technical replicates. Correlation (color key) indicates the Pearson correlation coefficient.
